# Cutaneous Involvement in Diseases with Plasma Cell Differentiation: Diagnostic Approach

**DOI:** 10.3390/curroncol29050246

**Published:** 2022-04-24

**Authors:** Magda Zanelli, Andrea Palicelli, Francesca Sanguedolce, Maurizio Zizzo, Alessandra Filosa, Linda Ricci, Camilla Cresta, Giovanni Martino, Alessandra Bisagni, Eleonora Zanetti, Francesco di Donato, Beatrice Melli, Alessandra Soriano, Luca Cimino, Alberto Cavazza, Lisa Francesca Vivian, Stefano Ascani

**Affiliations:** 1 Pathology Unit, Azienda USL-IRCCS di Reggio Emilia, 42123 Reggio Emilia, Italy; andrea.palicelli@ausl.re.it (A.P.); alessandra.bisagni@ausl.re.it (A.B.); eleonora.zanetti@ausl.re.it (E.Z.); alberto.cavazza@ausl.re.it (A.C.); 2 Pathology Unit, Policlinico Riuniti, University of Foggia, 71122 Foggia, Italy; francesca.sanguedolce@unifg.it; 3 Surgical Oncology Unit, Azienda USL-IRCCS di Reggio Emilia, 42123 Reggio Emilia, Italy; maurizio.zizzo@ausl.re.it; 4 Section of Pathological Anatomy, United Hospitals Ancona, 60126 Ancona, Italy; alessandra.filosa@ospedaliriuniti.marche.it; 5 Pathology Unit, Azienda Ospedaliera Santa Maria di Terni, University of Perugia, 05100 Terni, Italy; l.ricci@aospterni.it (L.R.); cresta.camilla@gmail.com (C.C.); g.martino1@studenti.unica.it (G.M.); s.ascani@aospterni.it (S.A.); 6 Department of Biomedical and Neuromotor Sciences, School of Anatomic Pathology, Bellaria Hospital, University of Bologna, 40126 Bologna, Italy; francesco.didonato6@studio.unibo.it; 7 Department of Obstetrics and Gynaecology, Fertility Center, Azienda USL-IRCCS di Reggio Emilia, 42123 Reggio Emilia, Italy; beatrice.melli@ausl.re.it; 8 Clinical and Experimental Medicine PhD Program, University of Modena and Reggio Emilia, 41124 Modena, Italy; 9 Gastroenterology Division, Azienda USL-IRCCS di Reggio Emilia, 42123 Reggio Emilia, Italy; alessandra.soriano@ausl.re.it; 10 Department of Surgery, Medicine, Dentistry and Morphological Sciences, University of Modena and Reggio Emilia, 41124 Modena, Italy; luca.cimino@ausl.re.it; 11 Ocular Immunology Unit, Azienda USL-IRCCS di Reggio Emilia, 42123 Reggio Emilia, Italy; 12 Pathology Unit, IRCCS Humanitas Research Hospital, 20089 Milan, Italy; lisa.vivian@humanitas.it

**Keywords:** plasma cell neoplasms, multiple myeloma, primary cutaneous marginal zone lymphoma, amyloidoma, plasmablastic lymphoma

## Abstract

Neoplasms with plasma cell differentiation may occasionally involve the skin. Cutaneous lesions may represent the first sign of an underlying systemic plasma cell malignancy, such as multiple myeloma, or the skin itself may be the primary site of occurrence of a hematological tumor with plasma cell differentiation. Starting from examples encountered in our daily practice, we discussed the diagnostic approach pathologists and clinicians should use when faced with cutaneous lesions with plasma cell differentiation. Cases of primary cutaneous marginal zone lymphoma, localized primary amyloidosis/amyloidoma, and cutaneous manifestations (secondary either to multiple myeloma or to plasmablastic lymphoma) are discussed, focusing on the importance of the adequate patient’s work-up and precise clinicopathological correlation to get to the correct diagnosis and appropriate treatment. The pertinent literature has been reviewed, and the clinical presentation, pathological findings, main differential diagnoses, treatment, and outcome of neoplasms with plasma cell differentiation involving the skin are discussed.

## 1. Introduction

The skin may be the primary site of occurrence of hematological malignancies, but skin lesions may also represent the first clue of an underlying systemic hematological disease. 

Tumors with plasma cell differentiation include a rather wide spectrum of disorders, which may rarely cause skin manifestations. A comprehensive clinical evaluation of the patient is critical for the precise pathological diagnosis and consequent appropriate management. 

This report aims to provide insights into the cutaneous manifestations observed in neoplasms with plasma cell differentiation. In our case study, we reported examples of primary cutaneous marginal zone lymphoma (PCMZL), localized primary amyloidosis/amyloidoma, and cutaneous manifestations (secondary either to multiple myeloma (MM) or to plasmablastic lymphoma (PBL)). The pertinent literature has been reviewed, and the clinical manifestations, pathological features, main diagnostic issues, treatment, and prognosis of these entities are discussed. The main clinic-pathological features of the reported cases are summarized in Table S1 (Supplementary Materials).

PCMZL is a low-grade, cutaneous B-cell lymphoma, and its rare plasmacytic variant consists almost entirely of a proliferation of neoplastic plasma cells [1,2,3,4,5,6,7,8,9,10,11,12,13,14,15,16,17,18,19,20,21]. Hence, the distinction of the plasmacytic variant of PCMZL, from skin involvement by MM, relies on the precise clinicopathological correlation. 

According to the European Organization for Research and Treatment of Cancer (EORTC)/World Health Organization (WHO) classification of primary cutaneous lymphoma, several cases reported in the past as primary cutaneous plasmacytoma either represent secondary cutaneous manifestations of an unrecognized MM or, in absence of an underlying MM, would be better re-classified as PCMZL, plasmacytic variant [1]. 

Localized primary amyloidosis, referred as amyloidoma or tumoral amyloidosis, is a rare form of amyloid deposition, presenting as a solitary mass that is usually mistaken as a neoplasm [1]. It has been described at almost any anatomic site, including the skin [22,23,24,25,26,27,28,29]. Localized primary amyloidosis generally follows a benign course, and systemic diseases with an aggressive behavior, such as MM and systemic amyloidosis, need to be ruled out. 

Despite uncommon, cutaneous involvement, MM may be the result of direct extension to the skin from underlying bone lesions [30,31,32,33,34,35,36,37,38,39] 

PBL is an aggressive lymphoma of B-cell origin, often occurring in the setting of immunosuppression (IS) and involving mainly extranodal sites [40,41,42,43,44,45,46,47]. Only a few cases of PBL limited to the skin have been reported so far, and skin involvement by PBL often develops, in the context of a systemic disease [48,49,50,51]. 

The goal of our study is to highlight that cutaneous lesions, characterized by neoplastic proliferation of plasma cells, should be always analyzed with a critical eye, and a proper patient’s workup is essential for a correct diagnosis. 

## 2. PCMZL

### 2.1. Clinicopathological Example of PCMZL, Plasmacytic Variant

A 77-year-old female presented with a solitary, non-pruritic, slightly infiltrated, erythematous papule 1 cm in diameter on her left breast. The patient had a history of breast cancer, treated with left breast quadrantectomy, several years before. Blood tests, including tumor markers (CA15-3 and CEA), were within normal limits, and no palpable adenopathy was clinically evident. The skin lesion had appeared 20 days before and, in the suspect of breast cancer recurrence, the lesion was surgically excised. 

**Figure 1:**
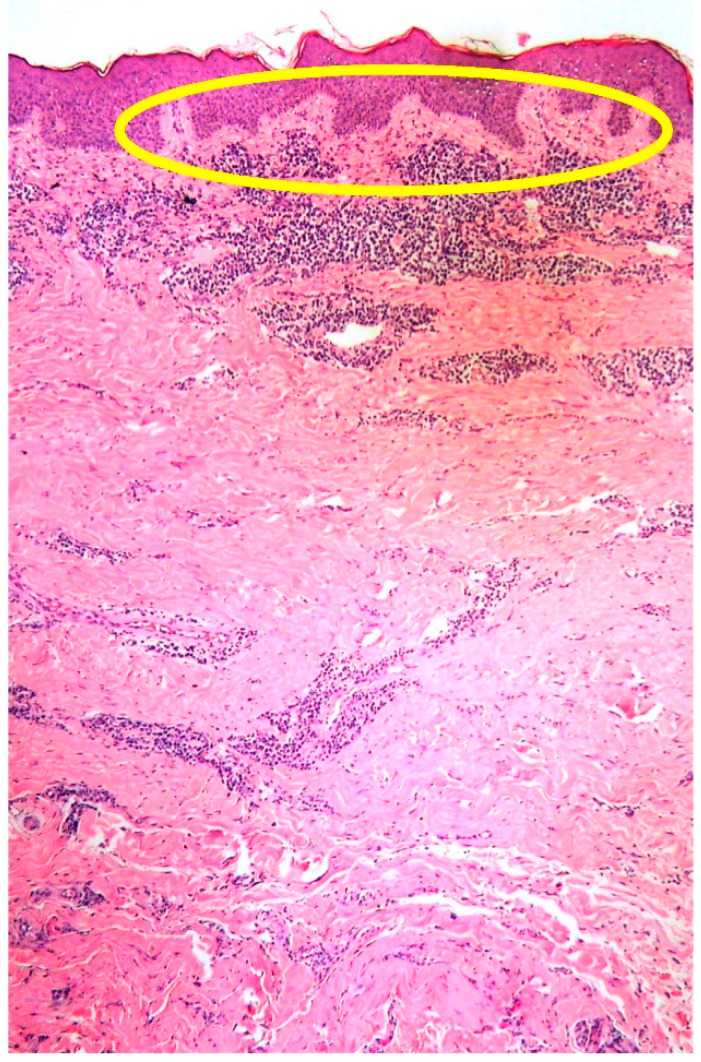
Low-power view of PCMZL, plasmacytic variant, showing a dermal, non-epidermotropic infiltrate (the lack of epidermotropism highlighted within the yellow circle) (HE, magnification 40×; original image from Prof. S.A.).

Histology showed a rather dense, non-epidermotropic, interstitial, and perivascular dermal infiltrate involving the superficial and profound dermis (Figure 1). 

**Figure 2:**
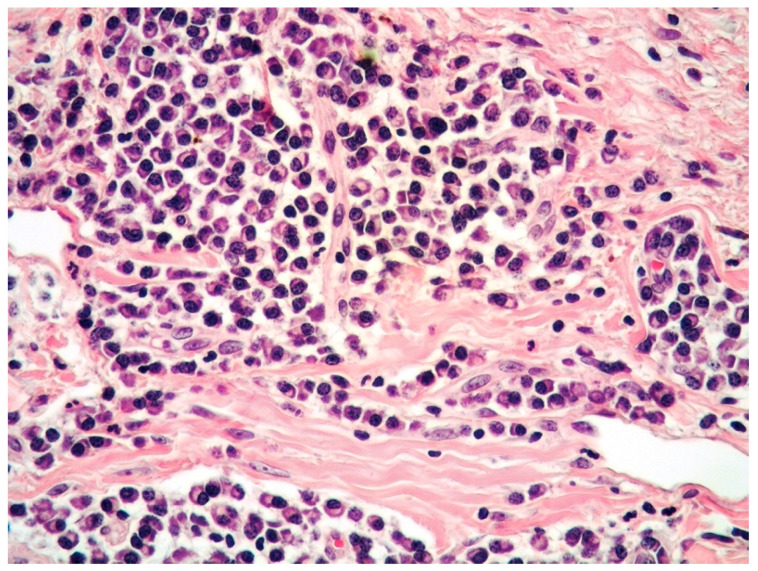
High-power view of PCMZL, plasmacytic variant, predominantly composed of mature plasma cells (HE, magnification 200×; original image from Prof. S.A.).

The infiltrate was mainly composed of mature-looking plasma cells with a minor component of small lymphocytes of B-cell origin (Figure 2).

**Figure 3:**
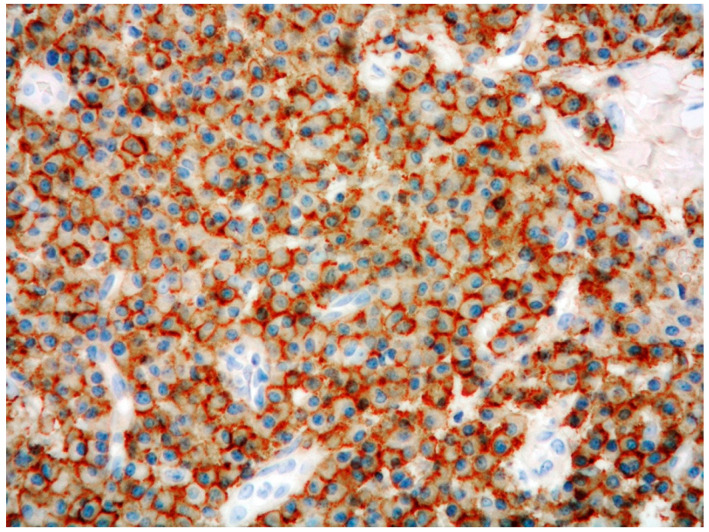
High-power view of PCMZL, plasmacytic variant: CD138 stain highlighting the plasma cell infiltrate (immunostaining, magnification 200×; original image from Prof. S.A.).

**Figure 4:**
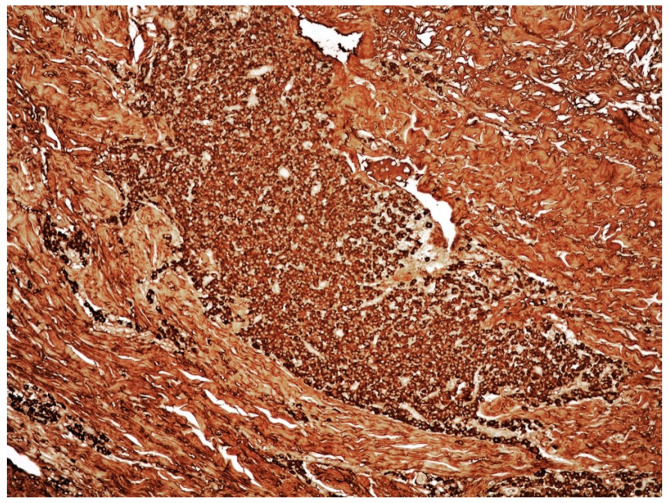
Medium-power view of PCMZL, plasmacytic variant: plasma cells elements monotypic for kappa immunoglobulin light chain (immunostaining, magnification 100×; original image from Prof. S.A.).

The plasma cells expressed plasma cell-related markers, such as CD138, and were monotypic for kappa immunoglobulin light chain (Figure 3 and Figure 4).

CD56, cyclin D1, IRTA1, IgG4, and human herpes virus 8 (HHV8), as well as both T- (CD3, CD4, and CD8) and B-cell markers (CD20 and PAX5), were negative in the plasma cell elements. The proliferative fraction was very low (Ki67: 5%). Clonal immunoglobulin heavy chain (IGH) rearrangement was identified. Cutaneous involvement of a plasma cell neoplasm was reported, stressing the need of a strict clinicopathological correlation to distinguish between secondary cutaneous involvement of MM and PCMZL. Bone marrow (BM) biopsy ruled out MM, whereas the presence of a small component of B lymphocytes admixed with the predominant plasma cell population tended to favor the diagnosis of PCMZL, plasmacytic variant, rather than primary cutaneous plasmacytoma. The patient was administered immunochemotherapy (four cycles of rituximab plus chlorambucil) and disease-free at 24 months from diagnosis.

### 2.2. Epidemiology, Clinical Features, Outcome and Treatment

Primary cutaneous B-cell lymphomas (PCBCLs) are a group of extranodal non-Hodgkin lymphomas, primarily involving the skin in the absence of extracutaneous spread at the time of diagnosis [1]. 

PCMZL is a low-grade lymphoma accounting for approximately 2–7% of all primary cutaneous lymphomas [1,2,3,4,5,6,7,8,9,10,11,12,13,14,15,16,17,18,19,20,21]. It was included in the group of extranodal marginal zone lymphoma of the mucosa-associated lymphoid tissue (MALT) in the last 2017 WHO classification of tumors of haematopoietic and lymphoid tissue [21]; whereas, in the 2018 update of the classification of cutaneous lymphomas by EORTC/WHO, PCMZL is actually considered a specific entity [1].

It often affects adults, with a median age of 55 years, presenting with reddish/violaceous solitary or multiple plaques or nodules, often localized on the trunk and arms [1,2,3,4,5]. Different microorganisms are considered to be implicated in the etiology of extranodal marginal zone lymphoma of MALT, such as *Helycobacter pylory* in gastric cases and *Clamydia psittaci* or *Clamydia jejuny* in ocular cases [21]. 

In PCMZL, a correlation with *Borrelia burgdorferi* has been observed in some studies, but not confirmed in others [2,7,8,9]. The outcome is excellent (5-year survival rate: 98%) [1,2,10,11]. Although recurrences are reported in 50% of cases, spread to extracutaneous sites is very rare, and transformation to high grade lymphoma represents a rare event, as well [12,13]. Surgery may be the treatment of choice for solitary lesions; radiotherapy and CD20 monoclonal therapy (Rituximab) are other therapeutic options, whereas antibiotic treatment is taken into consideration in cases associated with *Borrelia burgdorferi*. Chemotherapy is used in cases with extracutaneous spread [1,2,13,14].

### 2.3. PCMZL: Histopathological Variants

Cerroni describes five histopathological forms of PCMZL, i.e., the conventional, lymphoplasmacytic, plasmacytic, blastoid, and cutaneous amyloidoma variants [2].

The conventional form occurs frequently in adults and younger adults, affecting the trunk and upper extremities, whether the lymphoplasmacytic form is more common in the elderly and lower extremities are preferentially involved. The association with *Borrelia burgdorferi* seems more frequent in the lymphoplasmacytic form [2,7,8]. The architectural pattern of PCMZL consists of nodular or diffuse infiltrates within the dermis, often extending to the subcutis, with sparing of epidermis. 

Depending on the predominating cell types, the histological appearance may vary among the different variants. The neoplastic population in the conventional variant is much more polymorphic than in the other variants consisting of a combination of marginal zone cells with abundant clear cytoplasm, lymphoplasmacytoid lymphocytes, and plasma cells. Hence, a characteristic feature of the conventional type is the biphasic pattern of growth, with alternation of dark areas of reactive lymphocytes and sometimes with germinal centers and pale zones composed of marginal zone cells. Unlike the other variants, in the conventional form, reactive cells (histiocytes, eosinophils, and lymphocytes) often outnumber the neoplastic cells.

The lymphoplasmacytic variant has a monomorphous histology, with lymphoplasmacytoid lymphocytes being the predominating cells; additionally, plasma cells are often observed at the periphery of the infiltrate and numerous intranuclear inclusions, and so-called Dutcher bodies are seen. Most cases of lymphoplasmacytic variant were formerly named cutaneous immunocytoma [15].

The plasmacytic variant of PCMZL consists of a predominance of plasma cells, sometimes admixed with blastoid elements. Intracytoplasmic inclusions (so-called Russel bodies) are common, whereas Dutcher bodies are infrequent [16,17,18,19].

The blastoid variant may arise de novo or in patients with a previous diagnosis of PCMZL, and the behavior seems to be different in these two settings [20]. Cases arising as a blastoid variant from the beginning show a good outcome, similarly to the other variants of PCMZL, whereas cases arising in patients with a previous diagnosis of PCMZL behave more aggressively as transformed lymphomas. The blastoid variant is composed mainly of mid- and large-sized blastoid cells, often admixed with neoplastic plasma cells. Dutcher and Russel bodies are rare.

Cutaneous amyloidoma (see the following section on amyloidoma/nodular amyloidosis) is regarded by some authors as a variant of PCMZL, or related to this lymphoma, because recurrences of cutaneous amyloidoma may present with the histology of conventional PCMZL [2].

### 2.4. PCMZL: Immunohistochemical and Genetic Profile

Immunohistochemical analyses show that the neoplastic lymphoid cells are positive for B-cell markers (CD20 and CD79 alpha) and BCL2, as well as lacking BCL6, CD10, CD5, and cyclin D1 expression, allowing distinction from other B-cell lymphomas, such as follicle center lymphoma and mantle cell lymphoma.

Depending on the different subtypes of PCMZL, there is a variable number of plasma cells stained by specific markers (CD138 and CD38) and with monotypic expression of immunoglobulin light chains. A discrete number of reactive T lymphocytes is observed, in particular in the conventional variant of PCMZL. The proliferation rate is usually low, with the exception of the blastoid variant.

Up to 39% of the plasmacytic variant cases are found to be IgG4-positive, despite not being associated with systemic IgG4 disease [18,19].

Some studies have suggested the existence of two following subsets of PCMZL [22].

The most common heavy chain class-switched type express IgG, IgA, or IgE and lack the expression of the chemokine CXCR3, which is considered to be relevant for the homing of neoplastic B lymphocytes to MALT. These class-switched cases are often rich in reactive T lymphocytes; the neoplastic B-cell component is small, and neoplastic plasma cells are often found at the periphery of the infiltrate and features often observed in marginal zone lymphomas (MZLs), arising at other sites such as lymphoepithelial lesions, colonization of reactive germinal centers, or transformation to aggressive B-cell lymphomas, are absent.

The small subset of non-class-switched PCMZL consists of large aggregates of B lymphocytes expressing IgM and CXCR3; these cases have more features in common with MZLs arising at other extranodal sites. Some authors regard class-switched PCMZLs as clonal lymphoproliferative disorders, rather than overt lymphomas. 

Genetic analyses reveal the presence of monoclonal rearrangement of the Ig genes in the majority of PCMZLs. The t(11;18)(q21;q21), found in MZLs arising at other extranodal sites, is usually not detected in PCMZL, and the IGH/MALT1 t(14;18)(q32;q21) is a rather uncommon event. Methylation of DAPK1 and CDKN2A is frequent, whereas *MYD88* mutations are not found [1,2,3].

## 3. Cutaneous Nodular Amyloidosis/Amyloidoma

### 3.1. Clinicopathological Example of Cutaneous Amyloidoma

A 63-year-old male presented with a non-tender and soft mass in his right calf, which had been slowly growing over the last 10 years. Computerized tomography (CT) angiography showed a subcutaneous, exophytic mass, 5 cm in maximum diameter, with no signs of skeletal muscle infiltration. The mass had a dyshomogeneous density, with low-grade enhancement.

**Figure 5:**
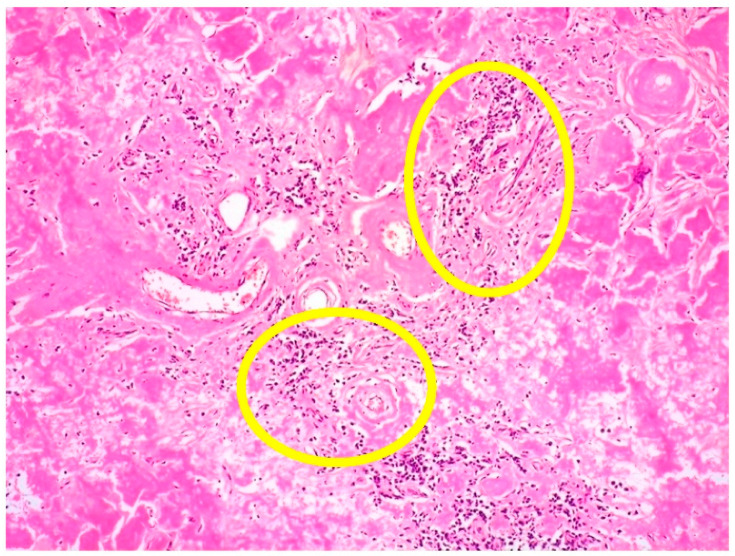
Cutaneous amyloidoma: amorphous and eosinophilic material, admixed with sparse plasma cells within dermis and subcutis (some foci of plasma cell infiltrate within yellow circles) (HE, 40× magnification; original image from Prof. S.A.).

Histologically, the incisional biopsy consisted of an abundant eosinophilic, amorphous material within the dermis and subcutis (Figure 5). 

**Figure 6:**
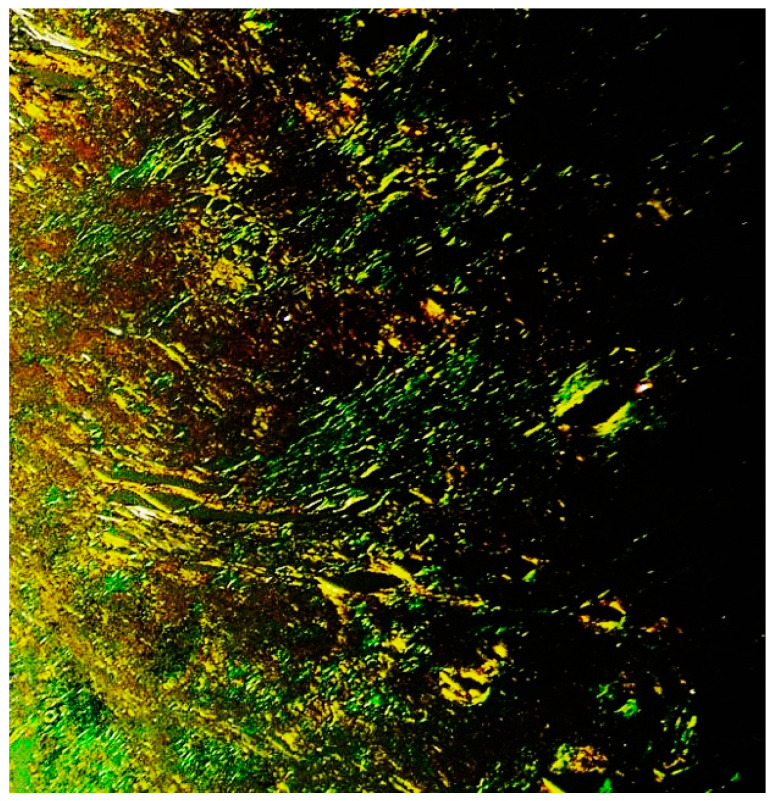
Cutaneous amyloidoma: typical green birefringence of the amorphous material (Congo red staining under polarized microscope, magnification 40×; original image from Prof. S.A.).

The material stained positive for Congo red, with green birefringence, under polarized light (Figure 6).

Some plasma cells, monotypic for the lambda light chain, were admixed. No serological monoclonal immunoglobulin was identified. Abdominal wall fat pad biopsy, BM biopsy, and total body CT scan showed no evidence of amyloid, plasma cell dyscrasia, or lymphoproliferative disorder. An amyloidoma was diagnosed and surgically excised. No further treatment was administered, and the patient is well, with no evidence of recurrence at 48 months after surgery.

### 3.2. Clinicopathological Features of Amyloid Deposits and Cutaneous Amyloidoma

The term amyloid applies to a homogeneous and eosinophilic material showing a typical green birefringence under polarized microscope, when stained with Congo red [23]. 

Amyloidosis refers to the extracellular deposition of amyloid, causing tissue damage. Amyloidosis represents a heterogeneous disorder with different forms of presentation and can be either systemic, the most common form, or localized. 

Amyloid is chemically composed of several types of proteins, usually associated with different diseases. The most common forms of amyloid are AL amyloid (derived from kappa and lambda immunoglobulin light chains, occurring in association with plasma cell and lymphoid disorders), AA amyloid (derived from serum amyloid A, occurring in the setting of chronic inflammation, infections, or neoplasms, other than lymphoid or plasma cell neoplasms), and beta-2-microglobulin amyloid (developing in long-term hemodialysis patients).

The least frequent presentation of *amyloid* deposition is as a localized mass, referred as amyloidoma or tumoral amyloidosis, and it is described at almost any anatomic site, including the skin [24,25,26,27,28,29]. Amyloidomas may develop either in the setting of lymphoproliferative disorders (AL amyloid) or in association with local trauma, infections, surgery, or chronic inflammation (AA amyloid).

Cutaneous amyloidoma/nodular amyloidosis consists of solitary or multiple waxy papules or nodules mostly localized on the legs, head, and neck region or the genital area [2,25,26,27,28]. Histologically cutaneous amyloidoma is made up of abundant amyloid deposition within dermis, with involvement of the vessel walls and often extending to subcutis. Additionally, sparse lymphocytes, plasma cells, and multinucleated giant cells are generally present; the plasma cells are often found to be monotypic. 

As previously mentioned, cutaneous amyloidoma is considered by some authors to be strictly linked with PCMZL, because recurrences of cutaneous amyloidoma may present with the histology of conventional PCMZL [2,26,27,28]. Cutaneous amyloidoma represents the cutaneous counterpart of nodular amyloidosis of the lung, which is considered to belong to the spectrum of MZL lymphoma [29]. Cutaneous amyloidoma often only requires surgery, and the outcome is usually favorable. 

## 4. Teaching Points of Case 1 (PCMZL) and Case 2 (Cutaneous Amyloidoma)

- PCMZL shows a spectrum of histopathological variants. The identification of small lymphocytes, marginal zone cells, and neoplastic plasma cells are helpful histological features for the diagnosis of classic cases of PCMZL. However, uncommon and atypical variants of PCMZL may be diagnostically challenging for pathologists. Case 1 represents an example of PCMZL, plasmacytic variant, characterized by an almost exclusive proliferation of neoplastic plasma cells.
- The plasmacytic variant of PCMZL is exceedingly rare, and the possibility of secondary cutaneous involvement by MM needs to be excluded through accurate staging procedures, including BM biopsy.
- The plasmacytic variant of PCMZL also raises the diagnostic possibility of primary cutaneous plasmacytoma. Extraosseous plasmacytoma is a plasma cell malignancy arising in patients with no evidence of MM. The majority of extraosseous plasmacytomas occurs in the upper respiratory tract. Currently, according to the WHO-EORTC classification, cases regarded in the past as primary cutaneous plasmacytoma need to be re-classified as PCMZL, plasmacytic variant.
- Cutaneous amyloidoma represents a rare manifestation of amyloidosis. The identification of amyloid deposition in the skin, as well in other body sites, imposes to rule out systemic diseases such systemic amyloidosis, lymphomas, and plasma cell dyscrasias, including MM.

## 5. MM

### 5.1. Clinicopathological Example of MM on Cutaneous Surgical Scar

A 75-year-old male presented with a solitary violaceous cutaneous nodule in the supraclavicular fossa at the site of a previous surgical scar. The patient had a 5-year history of MM treated, with the following lines of therapy: bortezomib, dexamethasone plus thalidomide, followed by autologous hematopoietic stem cell transplantation (auto-HSCT) as the first-line treatment; carfilzomib and dexamethasone as second-line treatment, 28 months after auto-HSCT; daratumumab, lenalidomide, and dexamethasome as third-line treatment. The skin lesion occurred during the administration of the third-line of therapy. 

**Figure 7:**
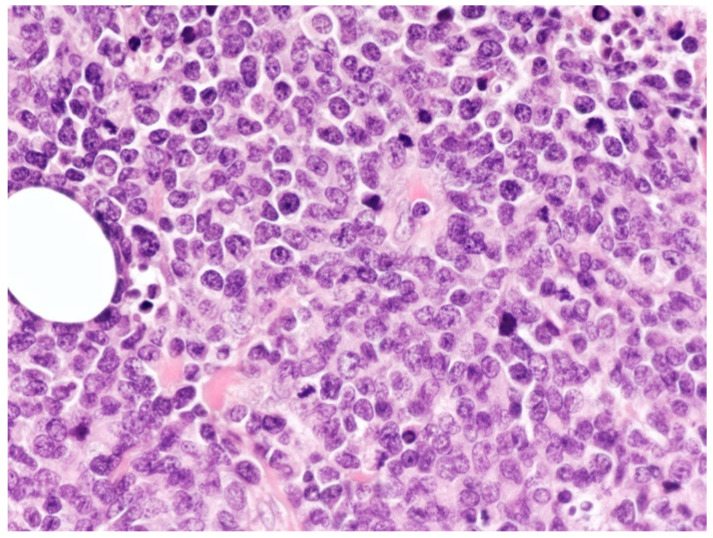
Cutaneous localization of MM: high-power view of the dense infiltrate, composed of large, atypical, polymorphic cells with a high nucleocytoplasmic ratio (HE, magnification 200×; original image from Prof. S.A.).

The histology of an incisional skin biopsy showed a dense infiltrate within the dermis and subcutis, composed of atypical, polymorphic cells (Figure 7). 

**Figure 8:**
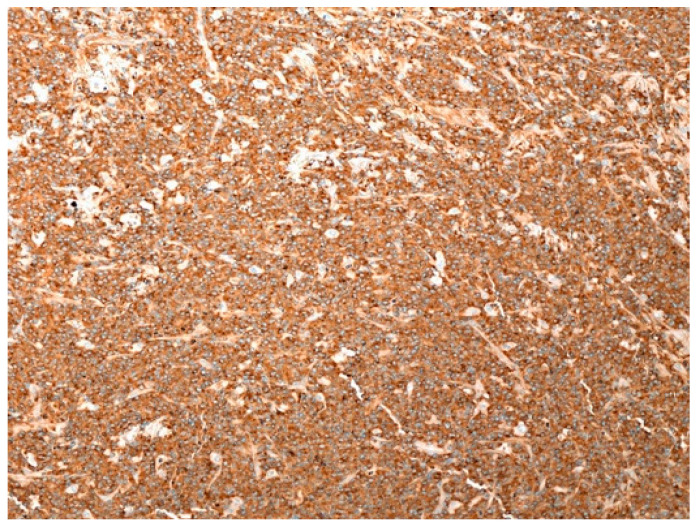
Cutaneous localization of MM: kappa immunoglobulin light chain restriction of the infiltrate (immunostaining, magnification 100×; original image from Prof. S.A.).

The cells were positive for CD138, MUM18, MUM1, and CD56, with partial expression of CD79 alpha and monotypic for kappa immunoglobulin light chain (Figure 8).

B- (CD20) and T-cell markers (CD3) were negative, as well as human herpes virus 8 (HHV8). The proliferative fraction was high (ki67: 90%).

EBV encoded ribonucleic acid (EBER) in situ hybridization was negative. A diagnosis of secondary cutaneous involvement by MM with polymorphic morphology was made. Subsequently, the patient was administered a fourth line of treatment (melphalan, followed by a second ASCT), with a good control of disease, and is still on follow-up.

### 5.2. Extramedullary Cutaneous Localization of MM: Clinical Features

MM accounts for more than 10% of all hematological malignancies. The disease affects adults of both sexes, often older than 50 years of age, with a median age of 70 years at diagnosis, but cases occurring in younger individuals are seen. The disease is characterized by a clonal proliferation of plasma cells, primarily confined to bone and BM, and it is responsible for the CRAB signs (hypercalcemia, renal dysfunction, anemia, and lytic bone lesions). Only in a minority (4%) of MM does neoplastic plasma cells involve sites outside the BM. Extramedullary involvement by MM is observed in the spleen, lymph nodes, and liver, which may be affected often in the advanced stages of the disease. 

Cutaneous involvement represents a rare complication of MM, described mainly in isolated case reports or small case series [30,31,32,33,34,35,36,37,38,39]. Hematogenous/lymphatic spread or dissemination from contiguous bone disease are considered the routes of spread to the skin. The exact mechanism behind the cutaneous migration of MM is not well-clarified. It has been supposed that biologically unique sub-clones that are capable of migrating to the skin may be selected, in particular, by high-dose chemotherapy used before ASCT. In a series from Korea of 1228 patients with MM, 1.14% of cases showed cutaneous involvement [33]. 

The largest multi-institutional retrospective study conducted so far identified 53 patients with cutaneous localization of MM [34]. Patients were more often males (60%), and the median age at skin involvement diagnosis was 63 years. The median time from MM diagnosis to skin involvement was 2.2 years. The median number of treatments prior to skin MM was 3. 

In patients with cutaneous involvement by MM, the most commonly found heavy chain immunoglobulin was IgG (40%), followed by IgA (36%) and IgD (1%), whereas the most frequent light chain was kappa (70%). 

Cutaneous involvement may be seen at any time in the course of the disease and not only during the late phases of disease.

**Figure 9:**
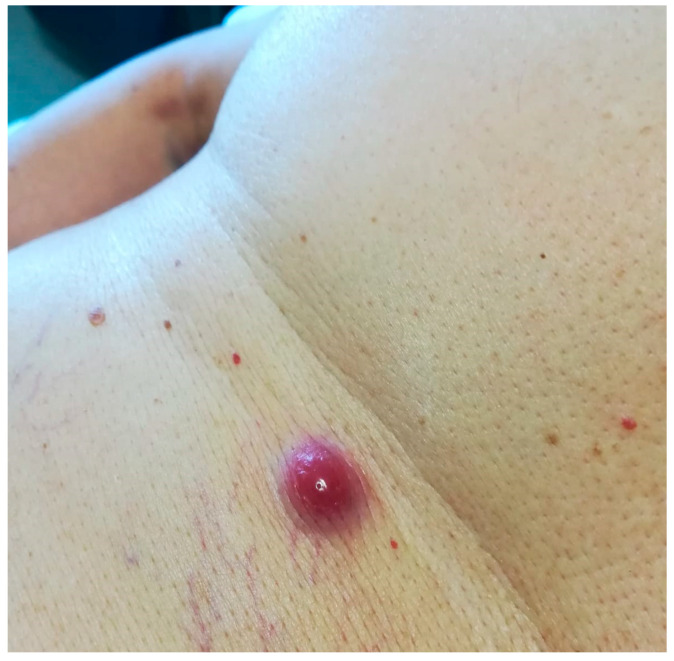
Cutaneous localization of MM: in vivo image of cutaneous purpuric nodule (original image from Prof. S.A).

Typically, the lesions present as solitary or multiple purpuric/brown papules or nodules of variable size, which may ulcerate (Figure 9).

In the study by Jurczyszyn et al., a third of patients had multiple lesions of more than 5 cm in diameter [34]. The most frequently involved sites were the chest, lower extremities, back, and buttocks. 

Analogously to other hematological neoplasms, such as myeloid leukemia, occasional cases of MM that localized to sites of previous cutaneous injuries have been reported (as our case 3), suggesting that trauma may play a role in skin homing by neoplastic plasma cells [35,36]. The median overall survival (OS) from time of skin MM diagnosis was of 8.5 months and a worse outcome is associated with IgA heavy chain disease and plasmablastic morphology. 

After the diagnosis of skin MM, almost all of the patients received different treatments: chemotherapy, proteasome inhibitors, immunomodulatory agents, radiotherapy, and HSCT. Novel agents, such as bortezomib, thalidomide, and lenalidomide, showed a transient efficacy in skin MM. Radiotherapy obtained partial and transient response [37,38].

### 5.3. Extramedullary Cutaneous Localization of MM: Histological Features

Histologically, the lesions consist of a proliferation of neoplastic plasma cells with two main histological patterns of growth: nodular and diffuse or, less frequently, interstitial, with the majority of cases showing a combination of the two patterns. The disease is based on the dermis, with sparing of the epidermis (so-called grenz zone) and often extending to the subcutaneous tissue. Amyloid deposition may be present within the vessel wall. 

The neoplastic plasma cells may have either a mature and recognizable morphology or, frequently, they show a higher degree of atypia with a polymorphic or plasmablastic morphology, difficult to recognize without appropriate immunohistochemical stains. Russel and Dutcher bodies are not generally observed. 

By immunohistochemistry, the neoplastic elements are positive for CD138, CD38, MUM1, and MUM18, as well as often for CD79alpha, CD56, and cyclin, with monotypic expression of immunoglobulin light chains. B-cell markers, such as CD20 and PAX5, are usually negative. Aberrant expression of T-cell markers (CD3 and CD4) has been rarely reported in MM, even in cases with skin involvement, and this feature may be misleading [39].

In the largest retrospective study by Jurczyszyn, a plasmablastic morphology was observed in 60% of skin MM, suggesting the aggressive behavior of skin MM [34]. Plasmablastic myeloma (PM) needs to be differentiated from PBL for therapeutic purposes (for differential diagnoses see following section on PBL).

## 6. PBL

### 6.1. Clinicopathological Example of Cutaneous Localization of PBL

A 40-year-old, HIV-positive male presented with a firm, nodular lesion in his right flank; the lesion had grown over a period of 2 weeks to a size of 3 cm in diameter. The skin overlying the nodule was thin and purple in color. His past medical records included multiple infections, such as atypical mycobacteria, pneumocystis, and cytomegalovirus. Eight months before, the patient had been diagnosed PBL with abdominal localization and had been administered chemotherapy, according to the modified COMP regimen (liposomal doxorubicin plus cyclophosphamide, without steroids because of his immunodeficiency condition and vincristine for the presence of peripheral neuropathy). Despite treatment, the disease had rapidly progressed; the cutaneous lesion developed approximately 2 weeks after the end of treatment. 

**Figure 10:**
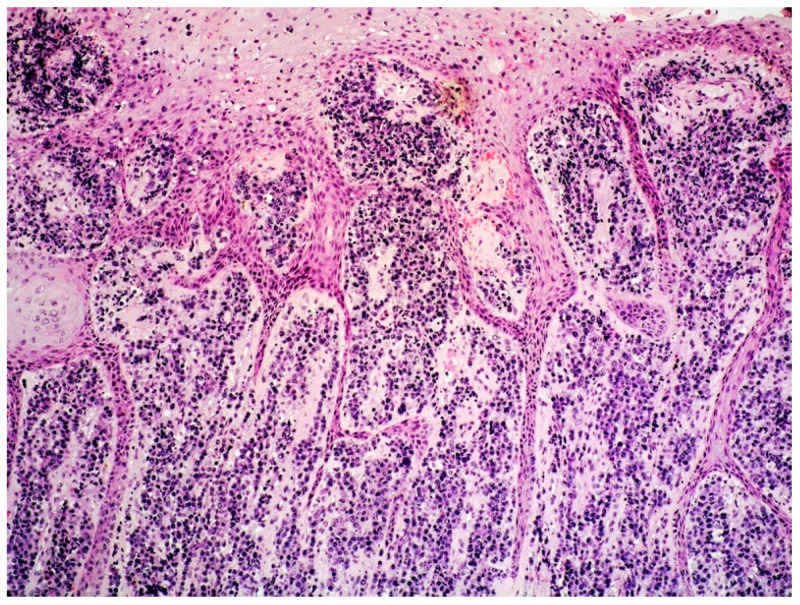
Cutaneous localization of PBL: medium-power view showing a dermal infiltrate lacking epidermotropic features and a hyperplastic epidermis (HE, magnification 40×; original image from Prof. SA).

The skin biopsy showed a hyperplastic epidermis overlying a diffuse infiltrate of large cells within the superficial and deep dermis, with no epidermotropism (Figure 10). 

**Figure 11:**
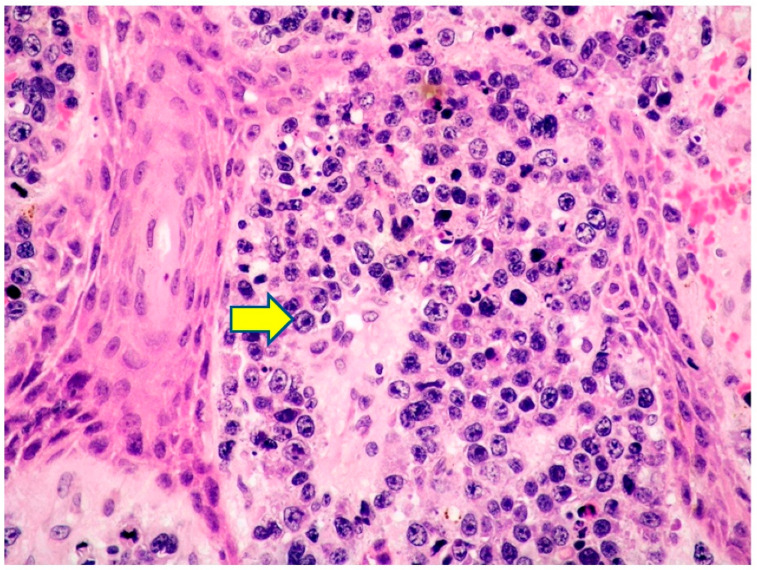
Cutaneous localization of PBL: high-power view highlighting the cytological details of the dermal neoplastic infiltrate, made up of large-sized cells with often evident nucleolus (yellow arrow pointing towards a large PBL cell with prominent nucleolus) (HE, magnification 200×; original image by Prof. S.A.).

The cells showed high nucleocytoplasmic ratio, a coarse chromatin pattern, and often prominent nucleolus. Mitotic figures were frequent (Figure 11). 

**Figure 12:**
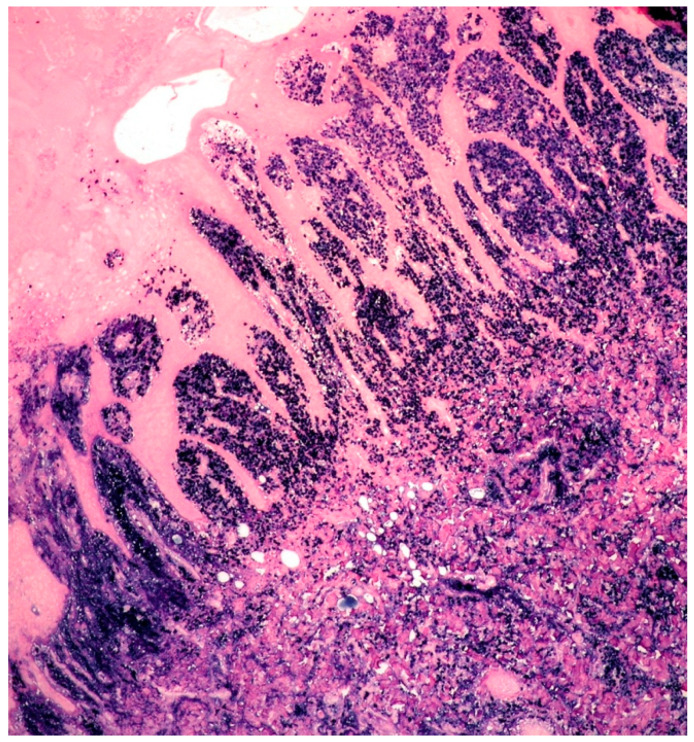
Cutaneous localization of PBL: diffuse EBV positivity, typically found in PBL and documented by EBER (in situ hybridization, magnification 40×; original image from Prof. S.A.).

The cells were weakly positive for CD45 and CD30 and strongly positive for CD138, MUM1, MUM18, and CD56, with kappa light chain restriction, indicating a degree of plasma cells differentiation. B-cell markers (CD20, CD79 alpha, and PAX5), T-cell markers (CD3 and CD5), as well as EMA, ALK1, CD10, and HHV8, were all negative. Diffuse Epstein Barr virus (EBV) positivity by EBV encoded ribonucleic acid (EBER) in situ hybridization was present (Figure 12).

The proliferative index was high (ki67: 90%). Skin involvement by PBL was diagnosed.

### 6.2. PBL: General Features

PBL, is a high-grade lymphoma, first described by Delecluse in 1997, and then recognized as a distinct entity in the 2008 WHO classification [40]. 

Initially described in HIV-positive individuals mainly in the jaw and oral cavity [40,41,42], it was subsequently recognized even in individuals with other forms of immunosuppression (IS), such as elderly individuals with age-related immune-senescence, patients with autoimmune diseases, transplanted patients, or in the setting of iatrogenic IS [42,43]. Immunocompetent individuals may rarely develop PBL [42,43]. 

The disease is prevalent in males (M:F = 4–5:1), usually occurring at a younger age in HIV-positive individuals (median 42 years), compared to patients with other conditions of IS (median 60) [42,43,44]. PBL generally involves extra-nodal sites, with the oral cavity and gastrointestinal tract being the most commonly affected sites [45,46,47]. 

The skin may rarely be affected by PBL, either primarily or in the context of a systemic disease [48,49,50,51]. EBV plays an important pathogenetic role in the occurrence of this aggressive lymphoma; in the setting of HIV-positive individuals, the majority of PBL is EBV positive, as documented by EBER [21]. 

Unfortunately, PBL prognosis remains poor, usually with a short survival (6–11 months), despite the introduction of intensive therapeutic schemes, such as hyper-CVAD-MA (hyperfractionated cyclophosphamide, vincristine, and doxorubicin plus dexamethasone alternating with methotrexate and cytarabine), CODOX-M/IVAC (cyclophosphamide, vincristine, doxorubicin, and methotrexate alternating with ifosfamide, etoposide and cytarabine), COMB (cyclophosphamide, oncovin, methyl-CCNU, and bleomycin), and EPOCH (etoposide, prednisone, vincristine, cyclophosphamide, and doxorubicin). Rituximab is not administered, due to common CD20 negativity of PBL [52,53,54,55]. Other treatments (anti CD30 brentuximab vedotin, bortezomib, and lenalidomide) and auto-HSCT [52,53,54,55] may be employed. Considering that high levels of PD-1 and PD-L1 expression have been found in PBL, possible therapeutic strategies may be the anti-PD-1 agents [56].

### 6.3. Cutaneous Localization of PBL: Clinical Features

Isolated cases of PBL with skin manifestations have been reported, so far [48,49,50,51]. Over half of cases (56%) with skin involvement occurred in HIV-positive individuals with a male predominance (68%). Apart from the strong association with HIV infection, the rest of the cutaneous PBL occurred mainly in transplanted patients, rare cases affecting individuals with autoimmune disorders, or the elderly, where age-related immunosenescence may have played a role. The lower limb was the site most frequently involved (50% of cases), and the disease was EBV-related in the majority of cases (80%). 

At the time of cutaneous manifestations, about half of cases had systemic involvement, whereas the other half presented with localized cutaneous PBL. 

Clinically cutaneous PBL presents as solitary or multiple grouped purple nodules or plaques, which often ulcerate.

### 6.4. PBL: Histology, Immunophenotype and Genetic Profile

PBL has a rather wide histological spectrum, from a monomorphous infiltrate of undifferentiated cells with plasmablastic, immunoblastic, or anaplastic features to a proliferation of more mature-looking plasma cells. Plasmablasts are typically large cells, with high nucleocytoplasmic ratio, often eccentrically placed nuclei, prominent nucleoli, and basophilic cytoplasm with perinuclear halo. Mitotic figures are often numerous, and necrosis may be observed. 

B-cell markers (CD20 and PAX5) are generally negative, as well as CD45, whereas CD79 alpha is positive in 40% of cases. Markers of plasma cell differentiation, such as CD138, MUM1/IRF4, CD38, VS38c, and PRDM1/BLIMP1, are often strongly positive, and neoplastic cells usually express monotypic cytoplasmic immunoglobulins. CD30 and EMA may be positive, and T-cell markers may be aberrantly expressed, causing a potential diagnostic pitfall with ALK-negative anaplastic large cell lymphoma (ALCL). CD56 may be expressed in 25% of cases. The proliferative index (Ki67) is usually high. EBV is found by EBER in a large number of cases (60–75%), particularly in HIV-positive and transplanted patients, whereas HHV8 is not documented in PBL. 

MYC protein overexpression is observed, even in cases lacking *MYC* translocations. This observation obviously suggests the possible existence of different mechanisms of *MYC* activation and protein expression. 

Clonal immunoglobulin heavy chain (IGH) rearrangement is generally observed. 

PBL etiology is strictly linked to the oncogenic activation of *MYC* and EBV infection is considered to play a relevant additional role. 

*MYC* rearrangement, generally with Ig gene partner, is found in about 50% of PBL cases and is usually associated with MYC protein overexpression [21,57]. *MYC* deletion and amplification are identified in a minority of cases (4% and 11%, respectively) [58]. 

### 6.5. PBL: Differential Diagnosis with Malignancies Showing Plasmablastic Features and Cutaneous Involvement

Differential diagnosis needs to take other aggressive hematological malignancies with a plasmablastic morphology into consideration, which may involve the skin, including PM, extracavitary primary effusion lymphoma (EC-PEL), EBV-positive diffuse large B-cell lymphoma, not otherwise specified (DLBCL, NOS), and anaplastic lymphoma kinase positive large B-cell lymphoma (ALK-positive LBCL) [21,45,59,60,61,62].

PM and PBL have a common morphology and immunophenotype, with expression of plasma cell markers. The clinical history is critical for this differential diagnosis. A positive EBV status, as well as a condition of IS, strongly support PBL diagnosis; the presence of lytic bone lesions, monoclonal paraproteinemia, hypercalcemia, renal dysfunction, and light chain proteins in urine, together with CD56 and cyclin-D1 positivity, are in favor of PM. However, it has to be mentioned that a definitive diagnosis cannot always be rendered, especially in cases with a solitary lesion, and, according to the current WHO, a diagnosis such as “plasmablastic neoplasm consistent with PBL or PM” may be made [21].

PEL, in its solid/extra-cavitary variant, more frequently involves lymph nodes, but may affects even extranodal sites, including the skin. Features in common with PBL are the frequent occurrence in the setting of IS, negativity for B-cell markers (although more often expressed in the extracavitary variant), and expression of plasma cell-related markers, CD30, and EMA. Unlike PBL usually expressing immunoglobulin, cytoplasmic immunoglobulin are often absent in PEL, although observed more frequently in EC-PEL. In PEL EBV infection is present mainly in the context of HIV-positive individuals. HHV8 expression is a required criterion for PEL diagnosis, allowing the distinction from PBL, which is HHV8 negative [21,60,61,62].

EBV-positive DLBCL, NOS is an EBV-driven, B-cell aggressive malignancy, initially described in patients over the age of 50 years, but subsequently identified even in younger adults. Both nodal and extra-nodal sites may be involved. Whereas younger patients generally have a nodal disease with a lower stage and better prognosis, and elderly individuals present more often have an extra-nodal disease, involving sites such as skin, lung, and gastrointestinal tract, and have a poor outcome [21,63,64,65]. The immunophenotype of EBV-positive DLBCL, NOS is different from PBL, showing a pan B-cell phenotype and lacking plasma cell-related markers.

ALK-positive LBCL is a rare, high-grade B-cell lymphoma with more frequent involvement of lymph nodes and mediastinum, although extranodal sites may be involved. Features in common with PBL are the plasmablastic/immunoblastic morphology and immunophenotypic profile with expression of plasma cell-associated antigens and EMA and often negativity for B-cell markers and CD45. The intrasinusoidal pattern of growth should make pathologists think of ALK-positive LBL. Then, the typical ALK protein expression, with a restricted granular cytoplasmic staining pattern, indicative of t(2;17)(p23;q23) or CTLC-ALK fusion protein, is the main diagnostic feature of this lymphoma type. Only a minority of cases, associated with NPM-ALK fusion protein, display a cytoplasmic, nuclear, and nucleolar ALK staining [21,59].

Additionally, ALK-positive LBCL is always EBV negative and often lacks CD30 expression, unlike PBL, which is frequently CD30- and EBV-positive.

PBL may show the aberrant expression of T-cell markers; this feature, in addition to CD30 expression, may cause the erroneous diagnosis of T-cell lymphoma.

## 7. Teaching Points of Case 3 (Cutaneous Localization of MM) and Case 4 (Cutaneous Localization of PBL)

- Once a clonal plasma cell proliferation is observed in the skin, it is mandatory to evaluate clinical, laboratory, and radiologic data, including BM biopsy, in order to exclude the presence of a systemic disease.
- Extramedullary involvement of the skin in MM is a rare event; it is associated with a poor prognosis and may occur at any stage of the disease.
- MM with skin localization may be poorly differentiated, often showing a polymorphic or plasmablastic morphology; hence, the plasma cell lineage may be difficult to recognize without appropriate immunohistochemical stains.
- PM needs to be differentiated from PBL, which usually occurs in patients with IS and is strongly associated with EBV, unlike PM.

## 8. Conclusions

Skin lesions, histologically composed of a neoplastic proliferation of plasma cells, need to be critically analyzed by pathologists and a comprehensive clinical evaluation of the patient is essential to establish if the skin is the primary site of occurrence of the neoplasm or the skin manifestation represents the first clue of a systemic disease. 

## Data Availability

The data presented in this study are available on request.

## Supplementary Materials

The following supporting information can be downloaded at: https://www.mdpi.com/article/10.3390/curroncol29050246/s1, Table S1: Clinic-pathologic features of 4 cases with skin involvement by neoplasms with plasma cell differentiation.
