# Supplementary material for: Cutaneous Involvement in Diseases with Plasma Cell Differentiation: Diagnostic Approach
Source: Curr Oncol. 2022 Apr 24;29(5):3026–43. doi: 10.3390/curroncol29050246 (PMC9139249; doi:10.3390/curroncol29050246)
Supplement: Supplementary file 1 [file curroncol-29-00246-s001.zip › curroncol-1676197-supplementary.pdf]

Table S1: Clinic-pathologic features of 4 cases with skin involvement by neoplasms with plasma cell differentiation

CASE AGE/SEX DIAGNOSIS	CLINICAL HISTORY	FEATURES OF SKIN LESIONS	HISTOLOGY	BM INVOLVEMENT	IHC HC ISH	MOLECULAR FEATURES	THERAPY OUTCOME
CASE 1<br>77/F<br>PCMZL	Skin lesion on breast (20 days history).<br>Previous breast cancer<br>No general symptoms<br>No adenopathy	Solitary, small, erythematous skin papule on breast	Non-epidermotropic interstitial/perivascular dermal infiltrate composed of mature plasma cells	negative	CD138+ Kappa+ lambda- CD20- PAX5- CD56- cyclin D1- IRTA1- IgG4- HHV8- CD3- CD4- CD8-<br>Ki67: 5%	IGH +	Rituximab plus chlorambucil (4 cycles);<br>Disease-free at 24 months
CASE 2<br>63/M<br>Amyloidoma	Mass of the calf (slowly growing over 10 years);<br>No signs of skeletal muscle infiltration ;<br>No general symptoms	Soft, non-tender, exophytic mass (5 cm) in the calf subcutis	Amorphous, eosinophilic material with sparse plasma cells within dermis and subcutis	negative	Amorphous material: Congo Red+<br>Plasma cells: CD138+ lambda + Kappa-	NP	Surgical excision only;<br>Disease-free at 56 months

CASE 3<br><br>75/M<br><br>Skin MM	5-year history of pluri-treated MM;<br>Skin lesion developed under the third line of MM therapy	Solitary, violaceous skin nodule in the supraclavicular fossa at site of a surgical scar	Dense infiltrate of large-sized atypical cells within dermis and subcutis with no epidermotropism	MM	CD138+ MUM18+ MUM1+ CD56+ CD79 alpha+/- kappa+ lambda- CD20- CD3- HHV8- EBER-<br>Ki67: 90%	NP	Melphalan plus second Auto-HSCT;<br>Good control of disease at 6 months
CASE 4<br>40/M<br><br>Skin PBL	HIV positivity;<br>PBL diagnosed 8 months before (abdominal mass);<br>Frequent infections;<br>Skin lesion developed 2 weeks after modified COMP CT	Solitary, firm, rapidly growing, violaceous skin nodule (3 cm) in the flank	Dense infiltrate of large-sized atypical cells within superficial and deep dermis, with no epidermotropism	NP at time of skin PBL	CD138+ MUM1+ MUM18+ CD56+ kappa+ lambda- CD45+/- CD30+/- CD20-<br>CD79 alpha-<br>PAX5- CD3- CD5- EMA- ALK1- CD10- HHV8- EBER+ Ki67: 90%	NP	No further treatment;<br>Exitus shortly after diagnosis of Skin PBL

LEGENDS: auto-HSCT: autologous hematopoietic stem cell transplantation; BM: bone marrow; CT; chemotherapy; modified COMP: liposomal doxorubicin plus cyclophosphamide; EBER: Epstein Barr virus encoded RNA; HC: histochemistry; IGH: immunoglobulin heavy chain; IIC: immunohistochemistry; ISH: in situ hybridization; MM: multiple myeloma; NP: not performed; PBL: plasmablastic lymphoma; PCMZL: primary cutaneous marginal zone lymphoma
